# Supplementary material for: Transcriptome Analysis Reveals Key Genes and Pathways Associated with the Regulation of Flowering Time in Cabbage (Brassica oleracea L. var. capitata)
Source: Plants (Basel). 2023 Sep 28;12(19):3413. doi: 10.3390/plants12193413 (PMC10574337; doi:10.3390/plants12193413)
Supplement: Supplementary file 1 [file plants-12-03413-s001.zip › Supplementary Materials.pdf]

Supplementary Information

Figure S1. Field investigation of flowering time in F_2 population.

Figure S2. Distribution of the expression levels of all genes in the twelve leaf samples. (a) The FPKM plotbox distribution of C491_VS_B602. (b) The FPKM density distribution of C491_VS_B602. (c) The FPKM plotbox distribution of F2-B_VS_F2-NB. (d) The FPKM density distribution of F2-B_VS_F2-NB.

Figure S3. Volcano plots of differentially expressed genes (DEGs) for each comparison in leaves of early- and late-bolting cabbage. (a) Volcano plots of differentially expressed genes (DEGs) for C491_VS_B602. (b) Volcano plots of differentially expressed genes (DEGs) for F2-B_VS_F2-NB.

Figure S4. Validate the expression patterns of *BoSEP2-1* and *BoSEP2-2* in other early-flowering (NCF8-1 and HC101-1) and late-flowering (NCF11-1 and HB9-1) materials using alternative samples.

Table S1. Overview of the transcriptome sequencing dataset.

Table S2. Go terms of 3748 DEGs common in the C491_VS_B602 and F2-B_VS_F2-NB comparison groups.

Table S3. KEGG pathways of 3748 DEGs common in the C491_VS_B602 and F2-B_VS_F2-NB comparison groups.

Table S4. Cabbage homologs of flowering-time genes.

Table S5. Information on 25 DEGs related to flowering time in C491_VS_B602 and F2-B_VS_F2-NB.

Table S6. Sequences of primer used for qRT–PCR in this study.
