# Supplementary figures and images for: Transcriptome Analysis Reveals Key Genes and Pathways Associated with the Regulation of Flowering Time in Cabbage (Brassica oleracea L. var. capitata)
Source: Plants (Basel). 2023 Sep 28;12(19):3413. doi: 10.3390/plants12193413 (PMC10574337; doi:10.3390/plants12193413)

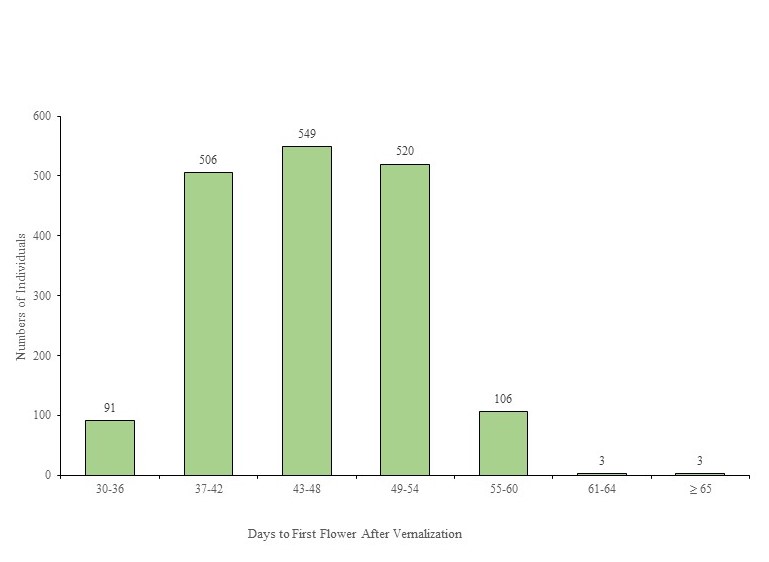

Supplement: Supplementary file 1 [file plants-12-03413-s001.zip › Figure S1.jpg]

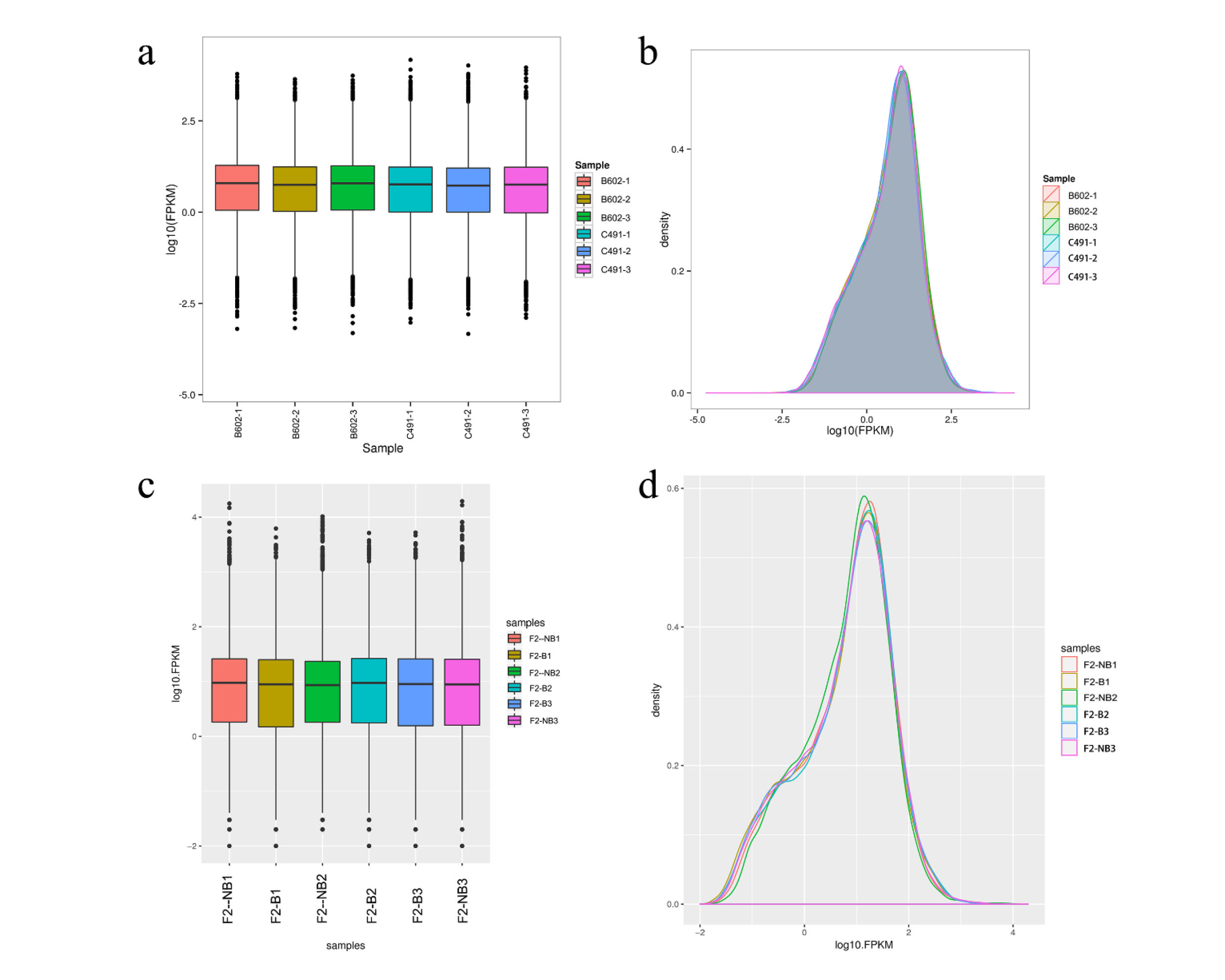

Supplement: Supplementary file 1 [file plants-12-03413-s001.zip › Figure S2.jpg]

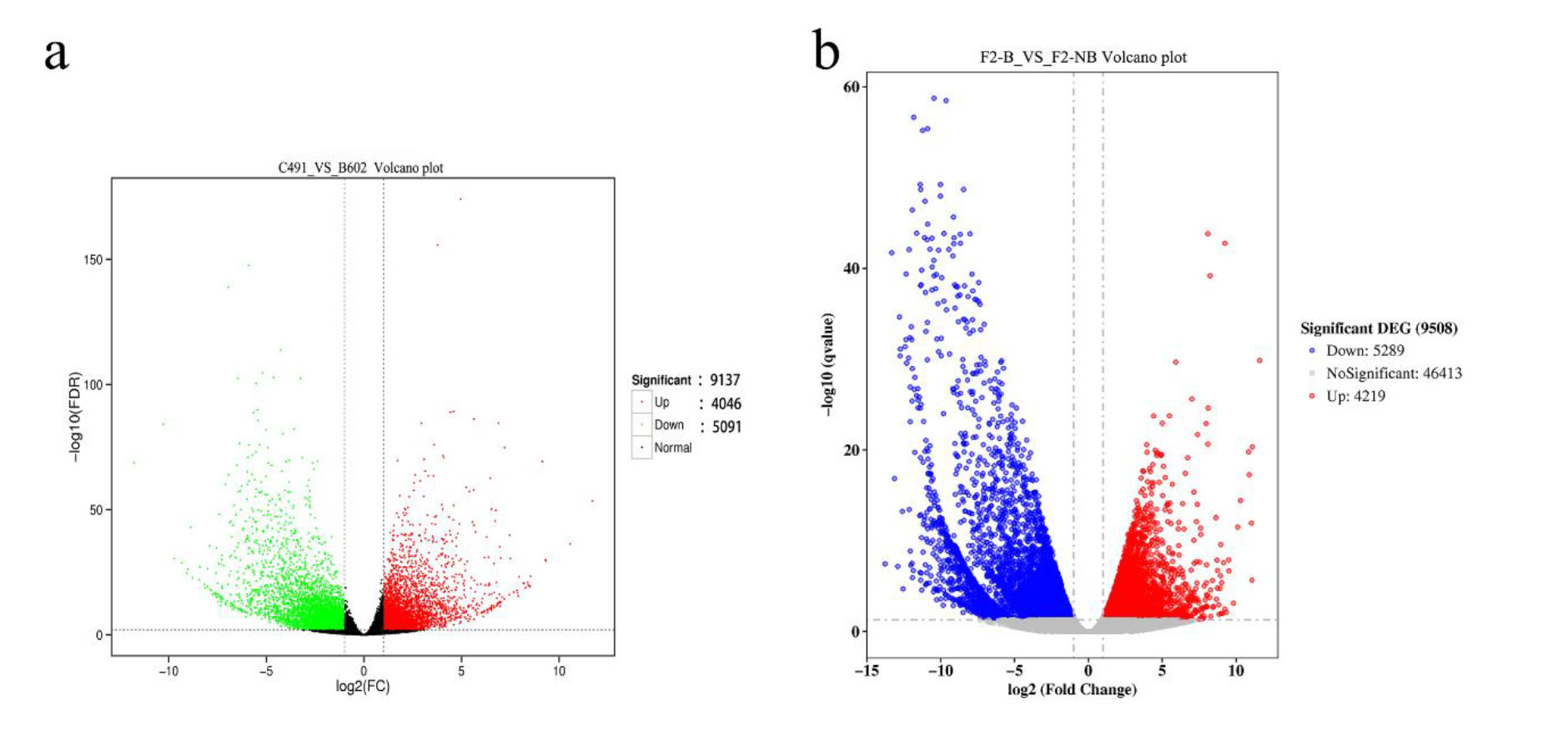

Supplement: Supplementary file 1 [file plants-12-03413-s001.zip › Figure S3.jpg]

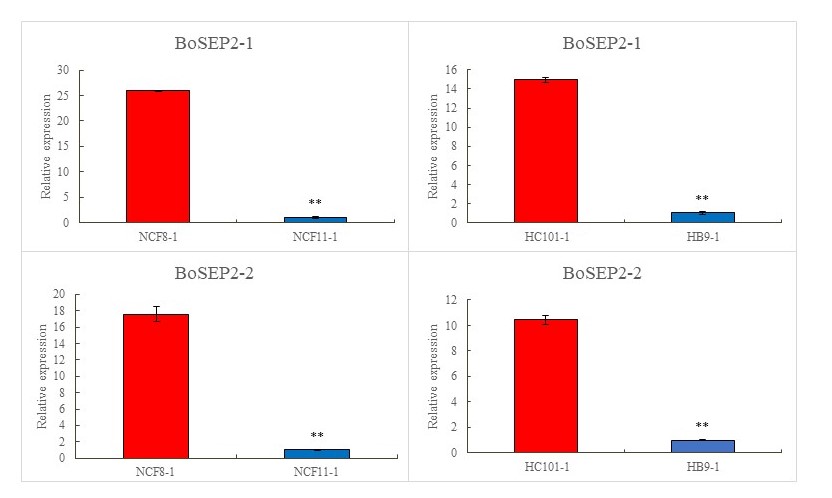

Supplement: Supplementary file 1 [file plants-12-03413-s001.zip › Figure S4.jpg]
